# Risks of habitat loss from seaweed cultivation within seagrass

**DOI:** 10.1073/pnas.2426971122

**Published:** 2025-02-18

**Authors:** Benjamin L. H. Jones, Johan S. Eklöf, Richard K. F. Unsworth, Lucy Coals, Marjolijn J. A. Christianen, Julian Clifton, Leanne C. Cullen-Unsworth, Maricela de la Torre-Castro, Nicole Esteban, Mark Huxham, Narriman S. Jiddawi, Len J. McKenzie, Masahiro Nakaoka, Lina M. Nordlund, Jillian L. S. Ooi, Anchana Prathep

**Affiliations:** ^a^Project Seagrass, Bridgend CF31 2AQ, United Kingdom; ^b^Department of Earth and Environment, Institute of Environment, Florida International University, Miami, FL 33199; ^c^Department of Ecology, Environment and Plant Sciences, Stockholm University, Stockholm SE-106 91, Sweden; ^d^Department of Biosciences, Swansea University, Swansea SA2 8PP, United Kingdom; ^e^School of Life and Environmental Sciences, Deakin University, Geelong, VIC 3216, Australia; ^f^Aquatic Ecology and Water Quality Management Group, Wageningen University and Research, Wageningen NL-6700 AA, The Netherlands; ^g^Curtin Institute for Energy Transition, Curtin University, Perth, WA 6102, Australia; ^h^Department of Physical Geography, Stockholm University, Stockholm SE-106 91, Sweden; ^i^School of Applied Sciences, Sighthill Campus, Edinburgh Napier University, Edinburgh EH11 4BN, United Kingdom; ^j^Institute of Marine Science, University of Dar Es Salaam, Zanzibar, Tanzania; ^k^Centre for Tropical Water and Aquatic Ecosystem Research, James Cook University, Cairns, QLD 4811, Australia; ^l^Field Science Center for Northern Biosphere, Hokkaido University, Akkeshi, Hokkaido 088-1113, Japan; ^m^Department of Earth Sciences, Natural Resources and Sustainable Development, Uppsala University, Uppsala SE-751 05, Sweden; ^n^Department of Geography Faculty of Arts and Social Sciences, Universiti Malaya, Kuala Lumpur 50603, Malaysia; ^o^Seaweed and Seagrass Research Unit, Division of Biological Sciences, Faculty of Science, Prince of Songkla University, Hat Yai 90110, Thailand

Seagrass meadows are thought to reduce water column marine bacterial pathogens, with new data from Fiorenza et al. (1) suggesting that this function extends to reducing disease in seaweed cultivation by 75%. As a result, Fiorenza et al. (1) advocate scaling seaweed production within seagrass meadows globally, highlighting benefits to local livelihoods. We argue that this is premature and dangerous for marine biodiversity and wider ecosystem functioning across the ~20.7 million km^2^ of suitable area. Fiorenza et al. (1) do not consider the holistic nature of the problem that they aim to provide solutions for nor the potential for complex unintended consequences (2, 3).

Water quality issues are globally prevalent. Understanding the role of seagrass in reducing pathogens, and how this facilitates and influences other ecological functions and services, is indeed highly important. However, suggestions made by Fiorenza et al. (1) are built on three flawed assumptions: first, that seaweed cultivation and seagrass can co-exist sustainably, two, that the results of their study are ubiquitous to the region, and, finally, that seaweed cultivation positively correlates with sustainable development.

First, despite historic and globally widespread seaweed cultivation, limited studies investigate effects on seagrass. In the few locations where studies exist, effects have been negative for seagrass structure and function and for associated biodiversity (4–7). We can only hypothesize the effects (e.g., displacement, entanglement) to seagrass-associated migratory species and megaherbivores that are also culturally significant for Indigenous people. Despite a potentially positive role of seagrass for seaweed production, the ecosystem services provided by seagrass, which are driven by structure, function, and biodiversity, likely suffer under cultivation scenarios.

Second, the results of the study do not provide a cause-and-effect relationship between seagrass and seaweed pathogen removal: 1) Fiorenza et al. (1) have not manipulated seagrass presence or conducted a more robust before-after control-impact study, and 2) have not actually measured seaweed pathogen presence or infections. In the best case, their data provide evidence to hypothesize a relationship that requires empirical experimental investigation.

Finally, in the context of sustainable development, seaweed cultivation strategies have mixed evidence for success (3, 8). Some evidence suggests that the activity worsens important development identifiers such as income and health, particularly for women (9, 10), and where it has been implemented to reduce fishing pressure, seaweed cultivation has instead increased (and often just displaced) fishing activity.

Understanding what trade-offs exist before advocating for large-scale expansion of this and other coastal industries is fundamental. Learning from the past, premature calls to scale oil palm production as a “vehicle to eradicate rural poverty” have been devastating for biodiversity and communities. Given the rapidly increasing threats faced by tropical marine habitats and their need to support coastal resilience to a changing climate, the risks posed by widespread seaweed cultivation to seagrass is high. This may also impact the most vulnerable in communities where the activity is prevalent (2). These complexities warrant further investigation to allow for a more nuanced discussion of short and long-term trade-offs before any scaling can occur.
